# The maximum contraceptive prevalence ‘demand curve’: guiding discussions on programmatic investments

**DOI:** 10.12688/gatesopenres.12780.1

**Published:** 2017-12-22

**Authors:** Michelle Weinberger, Emily Sonneveldt, John Stover

**Affiliations:** 1 Avenir Health, Glastonbury, CT, USA

**Keywords:** family planning, contraceptive use, demand, modelling, contraception

## Abstract

Most frameworks for family planning include both access and demand interventions. Understanding how these two are linked and when each should be prioritized is difficult. The maximum contraceptive prevalence ‘demand curve’ was created based on a relationship between the modern contraceptive prevalence rate (mCPR) and mean ideal number of children to allow for a quantitative assessment of the balance between access and demand interventions. The curve represents the maximum mCPR that is likely to be seen given fertility intentions and related norms and constructs that influence contraceptive use. The gap between a country’s mCPR and this maximum is referred to as the ‘potential use gap.’ This concept can be used by countries to prioritize access investments where the gap is large, and discuss implications for future contraceptive use where the gap is small. It is also used within the FP Goals model to ensure mCPR growth from access interventions does not exceed available demand.

## Introduction

FP Goals is a new model that leverages information about a country’s demographics, its current FP program, and global evidence on intervention effectiveness to quantify the impact of scaling up various interventions on future mCPR growth ( http://track20.org). When developing the model, there was a need to find a way to balance between ‘access’ and ‘demand.’ In a country with very little demand for family planning, large scale up of family planning services is likely to have limited impact and risks wasting resources (e.g. training providers on FP provision who have very few clients and lose their skills). However, in a country where there is a lot of unmet demand, scaling up services could have a large impact. Understanding this balance is important to ensure the model does not overestimate mCPR growth resulting from access based interventions (in the case where demand may be too low for all of the access to be utilized), but, even more importantly, it can help countries better understand and prioritize resources to ensure they have effective family planning programs.

Within the FP Goals model, the ‘demand curve’ is used mitigate projected growth from interventions with a direct impact on mCPR in cases where demand would be too low to allow the full growth to be realized. If projected mCPR growth is greater than what is allowable given demand, the model will limit mCPR growth. In addition, investments in SBC interventions can lower ideal number of children, thus increasing the maximum potential mCPR and easing the limiting factor over time. The demand curve can also be used as a stand-alone tool to inform discussions around balancing family planning investments.

## Methods: Developing the demand curve

### Looking to existing data

In order to include a balance between access and demand side interventions in the FP Goals model, a quantitative relationship using readily available data was needed. Data from all available DHS surveys was utilized ( https://statcompiler.com/en/), looking across a series of potentially relevant indicators. Scatterplots were developed comparing various indicators related to ‘demand’ to mCPR levels and mCPR growth rates. This was done separately for both married women and all women mCPR data.

The first indicator examined was ‘unmet need for contraception’, with the idea that one could allow the level of unmet need to determine how much further mCPR growth could be achieved without further investments in demand. However, the relationship between levels of unmet need and levels and changes in mCPR seen in the data did not allow this indicator to play the needed limiting role (e.g. there were large variations in mCPR at both low and high levels of unmet need). This is likely due to the complex nature of unmet need, the fact that it often increases before declining, and its reliance on information about pregnancy intention in a short time frame (next 2 years).

Next, the indicator ‘future intention to use’ was considered, with the idea of finding a relationship between future intention to use and subsequent mCPR growth to quantify how demand might limit or enable future mCPR growth. Future intention to use was calculated as the sum of the following individual categories: “intend to use in the future”, “In the next 12 months”, “Use later”, “Unsure about timing” as all are linked to a future intention to use (excludes responses of “unsure about use” and “does not intend to use”). But again, there was no clear relationship in the data that allowed for limiting mCPR growth based on levels of intention to use. Both unmet need and future intention to use are measures that directly look at contraceptive use, either a woman is not using despite not wanting to become pregnant (unmet need), or a women states that she plans to use contraception in the future. These two concepts both look at a rather short time frame, and, do not take wider societal influences and underlying norms into account.

Finally, an often overlooked indicator, ‘ideal number of children,’ was examined. When used, this indicator is often compared to actual fertility levels. However, for this purpose the interest was in how mean ideal number of children, on the aggregate, for a country related to total levels of modern contraceptive use in the country. While this indicator is a measure of fertility intentions, for the purposes of this analysis, the interest was to see to what extent the indicator could also signal where a country sits related to a spectrum of societal and individual constructs that sit behind intentions and motivations to use contraception.

**Figure 1.**
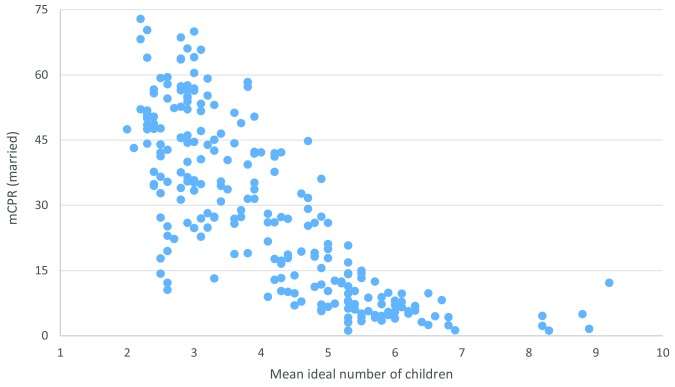
Scatterplot of mCPR married/in-union women against ideal number of children, all available DHS surveys.

When plotting these two indicators in a simple scatter plot, a surprising yet very clear relationship emerged for both married and all-women modern contraceptive use. Figure 1 shows the results for married/in-union women. In this graph, each blue dot represents a data point from a DHS survey (n=242). While there are large variations in levels of mCPR at any given level of mean ideal number of children, especially at lower levels of ideal number of children, there is a very clear maximum to the data. For example, at a mean ideal number of children between 6 and 6.5, mCPR for married/in-union has never gone above 9%, while, at a mean ideal number of children between 4 and 4.5, mCPR has never gone above 43%.

**Figure 2.**
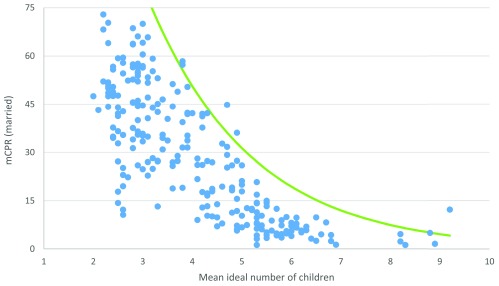
The Maximum Contraceptive Prevalence Demand Curve: Relationship between mCPR (married/in-union) and mean ideal number of children.

Building on this relationship, an exponential curve was fit to the *maximum* of this data in order to calculate the likely maximum mCPR at any given level of ideal number of children. This is illustrated in Figure 2, taking the same graph from the previous figure but now including the curve fit to the data in orange (y = 345*e ). This line was established by fitting a curve that best touched on the maximum mCPR values seen from the data at each level of ideal number of children.

An outlier point appears in the bottom right corner of this graph- this is Niger. In Niger, many women give very high numeric responses to the question on their ideal number of children (6% of respondents gave numbers ranging from 15–30 children), rather than non-numeric responses (only 3%). In other countries, much higher rates of non-numeric responses (and fewer high numeric responses) are seen. This pattern in Niger pulls up the mean for the country, making it an outlier from the other data.

A similar curve has been fit to mCPR for all women, while the same general pattern held among all women, the shape and level of the curve was slightly different (y = 185*e ). However, this curve may be less applicable, especially in countries with high levels of contraceptive use outside of marriage as this use contributes to the mCPR, but, is generally pre-childbearing so does not directly impact on fertility intentions. The highest recorded levels of mCPR are reached at an ideal level of children around three on both curves, therefore, the curve is cutoff at this point, recognizing that below this point, low fertility intentions are not limiting contraceptive uptake. Therefore, this concept is most applicable in countries with higher levels of ideal fertility.

## Results

### Examining time trends

**Figure 3.**
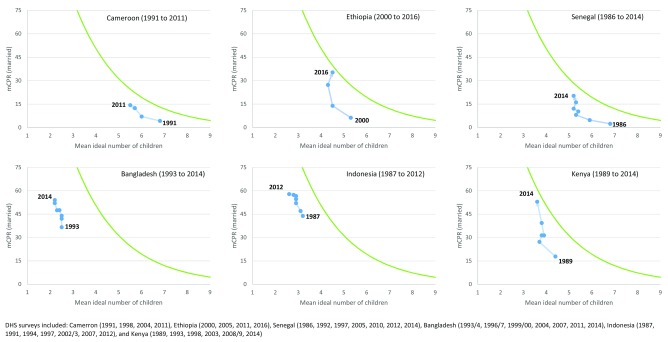
Demand Curve with time trends for 6 selected countries.

For countries that have had multiple DHS surveys, it is possible to look at how both mCPR and ideal number of children have changed over time. This is helpful to better understand how these two indicators change in relation to one another and how country patterns compare to the demand curve. Figure 3 shows DHS data for six countries (Cameroon, Ethiopia, Senegal, Bangladesh, Indonesia, and Kenya) plotted against the demand curve for married women. In this graph, each blue dot represents a DHS survey, the total span of years covered by the data is shown in each graph. Generally, it can be seen that increases in contraceptive use are coupled with declines in ideal number of children (movement from right to left on the graphs). However, in some instances, when the country was sitting well below the curve, mCPR growth was achieved without further changes in demand. This is evident by the vertical trend in the blue dots seen in Ethiopia, Senegal, and Kenya, and suggests that if enough demand exists (e.g. a wide enough gap between the country data point and the curve), there is room to increase mCPR without further changes in demand. However, the vertical changes seen in these countries indicate that mCPR increased without any shifts in fertility intentions meaning that the countries may face further limits to growth as they have now come close to the curve.

In general, the slope of the trend formed by the survey data closely mirrors the shape of the demand curve—there are slower changes in mCPR when ideal number of children remains high, and more rapid changes as countries shift towards lower levels of ideal number of children. For Indonesia and Bangladesh, data is only available from a time when ideal number of children was already low, so we are unable to see the full progression made by these countries. These countries both sit below an ideal number of children of 3, reflecting that, in both countries, fertility intentions are not a constraint on mCPR growth.

### Interpreting the demand curve

The curve represents the likely maximum mCPR that could be reached in a country given their level of demand. The gap between where a country sits (their blue dot) and the curve is referred to as the ‘potential use gap’- an estimate of the *maximum* mCPR growth that a country could expect to achieve within current levels of demand. While the curve is constructed using ideal number of children, an indicator that measures fertility desires, it is representing a wider set of social constructs that may be influencing the motivation to use, or not use, contraception. As noted earlier, the ‘demand curve’ concept is most applicable for countries with higher fertility intentions, and in fact, for countries with an ideal number of children below 3 there is no curve as in these countries it is assumed that fertility intentions are not limiting mCPR growth. There could be other factors limiting growth in these countries related to both access to services, and, knowledge and information about contraceptives. Rather, in these contexts, underlying social norms related to fertility desires and family size are likely not playing a limiting role.

If a country sits near to or above the line, the implication is that future growth in mCPR may be limited without further changes in demand. This could indicate a need to prioritize interventions that address underlying social norms in these countries, or, at a minimum, to set realistic expectations about future growth given the context. Looking back at Figure 3, this can be seen in the early data points in Cameroon, Ethiopia, and Senegal where each country moved to the left (e.g. lower ideal number of children)- in all three cases this change was coupled with little or moderate increases in mCPR. However, once at a higher level of demand, both Ethiopia and Senegal demonstrated more rapid increases in mCPR.

Changing levels of demand requires intensive interventions aimed at addressing a wide range societal norms, and not just awareness and knowledge of contraception. However, it is important to note that the implication of this analysis is not that interventions should directly address issues related to fertility intention- but rather, should deal with a wide-range of issues related to norms around fertility, family formation, and contraceptive use. This follows the latest thinking on Social and Behavior Change (SBC), which is aimed at addressing individual behavior as well as shifting social norms ^1^. While addressing underlying societal norms can be difficult, there is emerging evidence that well designed programs can impact these norms. For example, according to findings from the Nigerian Urban Reproductive Health Initiative (NURHI) ^2^ program exposure “was associated with improved ideation among women... and more positive ideation was associated with greater contraceptive use.” ^2^ The intervention also led to changes in the indicator of interest for this analysis- there was a statistically significant change in the ‘percent of married or cohabitant women who indicated wanting families of 3 or fewer children’ ^2^ – suggesting that this indicator can be used to signal wider issues related to societal constructs that may be influencing contraceptive use.

If a country sits well below the curve (e.g. has a large ‘potential use gap’), access investments alone could help to increase mCPR. This can be seen from the vertical increase (mCPR increased without changes in ideal number of children) seen in Ethiopia, Kenya and Senegal in Figure 3. However, even for countries sitting well below the demand curve, demand generation interventions may be needed to address barriers that are keeping modern contraceptive use low relative to what might be expected or needed for women to realize their fertility intentions. The focus of these interventions may be different- addressing gaps in knowledge, myths and misconceptions, and other more immediate barriers to use than for countries sitting near to the curve. For countries at lower levels of ideal number of children, an mCPR that falls below the maximum of the curve could indicate the existence of barriers to contraceptive use, or, could also indicate that other fertility determinants are being used to regulate fertility, meaning women are able to realize their fertility intentions without higher levels of contraceptive use.

It is worth noting that the line represents a maximum—it is likely that many countries will never actually be able to reach the line. Rather, this concept is meant to be a tool to help countries, especially those with high fertility intentions (as used elsewhere), think about where they might need to prioritize focus on increasing demand and addressing underlying social norms before seeing further mCPR growth, and where investments in access could be effective. In addition, because societal norms are often slow to change, this concept can be used to help manage expectations about future growth in countries that sit very near to the curve.

Making investments in access and demand is not an either/or choice; in-fact within the FP Goals model for some access interventions, including an element of demand generation (such as complementing Community Health Workers (CHWs) with comprehensive community engagement activities) results in additional impact, a fact proven out in the literature that sits behind the model’s impact matrix ^3^.

### Overview of global results

**Figure 4.**
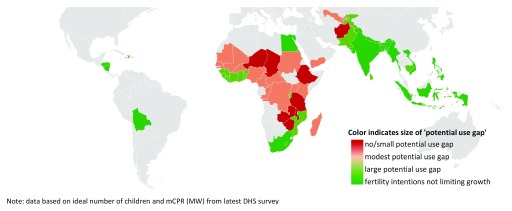
Countries colored based on size of potential use gap.

The map in Figure 4 shows countries based on the size of their potential use gap (red = very small gap, green = large gap/demand not limiting growth). This gives an indication of where levels of demand might limit further mCPR growth (red areas) and where investments in access alone could effectively drive more growth (light and dark green areas). This map is based on data from the latest DHS survey in each. Results are shown for all FP2020 countries with available data; the detailed data used to create this map can be found in Table 1. This is a first step to help think about how to get the balance between supply and demand right in countries. As can be seen, particularly in parts of Western and Central sub-Saharan Africa, the potential use gaps are very small. Further investments in demand side interventions, especially those that focus on changing underlying social norms, will be important to see further progress in mCPR growth in these countries. For countries with small gaps in Eastern and Southern Africa, this may reflect recent success in increasing mCPR that were not coupled with shifts in fertility intentions (as was shown for Ethiopia in Figure 3).

**Table 1.** Data from the 69 FP2020 countries on ideal number of children, mCPR, and potential use gap (e.g. additional mCPR growth within levels of demand) from latest survey in each country. This table looks at the potential use gap at the time of the latest survey in each country. For countries with older surveys, the calculated gap might not reflect the current situation. For countries where the gap is negative (Niger, Zambia, Zimbabwe) it suggests that these countries are slight outliers relative to the global curve, but, as they sit very near to the curve, further mCPR growth may be limited without further changes in demand.

Country	Source	Ideal # Children	mCPR (MW)	Maximum mCPR	Potential Use Gap	Interpretation
Afghanistan	2015 DHS	5.6	19.8	23.5	3.7	no or a small potential use gap
Bangladesh	2014 DHS	2.2	54.1	n/a	n/a	fertility intentions not limiting growth
Benin	2011–12 DHS	4.6	7.9	37.9	30.0	large potential use gap
Bolivia	2008 DHS	2.4	34.6	n/a	n/a	fertility intentions not limiting growth
Burkina Faso	2010 DHS	5.5	15	24.6	9.6	modest potential use gap
Burundi	2010 DHS	4.2	17.7	45.9	28.2	large potential use gap
Cambodia	2014 DHS	3.1	38.8	77.9	39.1	large potential use gap
Cameroon	2011 DHS	5.5	14.4	24.6	10.2	modest potential use gap
Central African Republic	1994–95 DHS	6.4	3.2	16.0	12.8	modest potential use gap
Chad	2014–15 DHS	8.2	5	6.7	1.7	no or a small potential use gap
Comoros	2012 DHS	5.3	14.2	27.1	12.9	modest potential use gap
Congo	2011–12 DHS	5	20	31.3	11.3	modest potential use gap
DR Congo	2013–14 DHS	6.1	7.8	18.5	10.7	modest potential use gap
Côte d'Ivoire	2011–12 DHS	5.2	12.5	28.4	15.9	large potential use gap
Egypt	2014 DHS	3	56.9	81.7	24.8	fertility intentions not limiting growth
Eritrea	2002 DHS	5.8	7.3	21.3	14.0	modest potential use gap
Ethiopia	2016 DHS	4.5	35.3	39.8	4.5	no or a small potential use gap
Gambia	2013 DHS	6	8.1	19.4	11.3	modest potential use gap
Ghana	2014 DHS	4.3	22.2	43.8	21.6	large potential use gap
Guinea	2012 DHS	5.8	4.6	21.3	16.7	large potential use gap
Haiti	2012 DHS	2.8	31.3	n/a	n/a	fertility intentions not limiting growth
Honduras	2011–12 DHS	2.8	63.8	n/a	n/a	fertility intentions not limiting growth
India	2005–06 DHS	2.3	48.5	n/a	n/a	fertility intentions not limiting growth
Indonesia	2012 DHS	2.6	57.9	n/a	n/a	fertility intentions not limiting growth
Kenya	2014 DHS	3.6	53.2	61.3	8.1	modest potential use gap
Kyrgyzstan	2012 DHS	3.9	33.7	53.1	19.4	large potential use gap
Lesotho	2014 DHS	2.6	59.8	n/a	n/a	fertility intentions not limiting growth
Liberia	2013 DHS	4.8	19.1	34.5	15.4	large potential use gap
Madagascar	2008–09 DHS	4.7	29.2	36.1	6.9	modest potential use gap
Malawi	2015–16 DHS	3.7	58.1	58.4	0.3	no or a small potential use gap
Mali	2012–13 DHS	5.9	9.9	20.3	10.4	modest potential use gap
Mauritania	2000–01 DHS	6.2	5.1	17.6	12.5	modest potential use gap
Mozambique	2011 DHS	4.8	11.3	34.5	23.2	large potential use gap
Myanmar	2015–16 DHS	2.5	51.3	n/a	n/a	fertility intentions not limiting growth
Nepal	2011 DHS	2.1	43.2	n/a	n/a	fertility intentions not limiting growth
Nicaragua	2001 DHS	2.9	66.1	n/a	n/a	fertility intentions not limiting growth
Niger	2012 DHS	9.2	12.2	4.2	-8.0	no or a small potential use gap
Nigeria	2013 DHS	6.5	9.8	15.2	5.4	modest potential use gap
Pakistan	2012–13 DHS	4.1	26.1	48.2	22.1	large potential use gap
Philippines	2013 DHS	2.8	37.6	n/a	n/a	fertility intentions not limiting growth
Rwanda	2014–15 DHS	3.4	47.5	67.5	20.0	large potential use gap
Sao Tome and Principe	2008–09 DHS	3.5	33.7	64.3	30.6	large potential use gap
Senegal	2014 DHS	5.2	20.3	28.4	8.1	modest potential use gap
Sierra Leone	2013 DHS	4.9	15.6	32.8	17.2	large potential use gap
Solomon Islands	2007 DHS	3.3	27.3	70.8	43.5	large potential use gap
South Africa	1998 DHS	2.9	55.1	n/a	n/a	fertility intentions not limiting growth
Sri Lanka	1987 DHS	3.1	40.6	77.9	37.3	large potential use gap
Sudan	1989–90 DHS	5.9	5.5	20.3	14.8	modest potential use gap
Tajikistan	2012 DHS	3.6	25.8	61.3	35.5	large potential use gap
Tanzania	2015–16 DHS	4.7	32	36.1	4.1	no or a small potential use gap
Timor-Leste	2009–10 DHS	5	21.1	31.3	10.2	modest potential use gap
Togo	2013–14 DHS	4.3	17.3	43.8	26.5	large potential use gap
Uganda	2011 DHS	4.8	26	34.5	8.5	modest potential use gap
Uzbekistan	1996 DHS	3.6	51.3	61.3	10.0	modest potential use gap
Vietnam	2002 DHS	2.4	56.7	n/a	n/a	fertility intentions not limiting growth
Yemen	2013 DHS	4.3	29.2	43.8	14.6	modest potential use gap
Zambia	2013–14 DHS	4.7	44.8	36.1	-8.7	no or a small potential use gap
Zimbabwe	2015 DHS	3.9	65.8	53.1	-12.7	no or a small potential use gap

## Discussion

**Figure 5.**
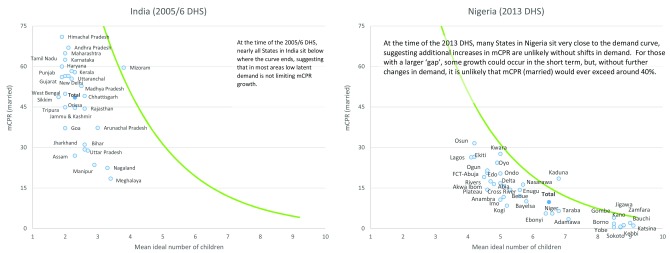
Illustrative example of sub-national demand curves in India (left) and Nigeria (right).

As a standalone concept, the demand curve can be used to help countries make a preliminary assessment as to future growth that can be expected given existing levels of demand. This can be used to advocate for prioritization of interventions that address underlying social norms, and, to manage expectations about future growth in contexts where the potential use gap is small. Because DHS data allows the mean ideal number of children to be calculated by sub-national areas, the graph can be replicated for States or Regions, or, by socio-economic status (e.g. wealth quintile, education) to see if there are specific areas where a small potential use gap may hinder future growth in contraceptive use. As can be seen in the examples in Figure 5 the national data often hides wide variation at the sub-national level. The more detailed look into this data can help inform strategic discussions and planning, allowing prioritization of different types of interventions in different sub-national areas.

Because this concept it built into the FP Goals model, a full application of the model can allow for a more refined analysis not only of where demand interventions are needed, but, to what degree scaling up SBC interventions can effectively create additional demand. Within the context of a full model application, this underlying concept allows for strategic discussions in country about the right mix of access and demand investments.

## Conclusion

The maximum contraceptive prevalence ‘demand curve’ provides a simple way to contextualize thinking about the balance in investments between access-focused interventions and demand side interventions. It can be used to stimulate discussions at both the global and country level. It is useful to provide a cursory look into this area, and can be supplemented with further analysis and use of other existing concepts (e.g. proximate determinants model).

The ‘demand curve’ can be used by countries to prioritize access investments in areas where the gap is large and discuss implications for future contraceptive use uptake in areas where the gap is small. Within the FP Goals model, this relationship mitigates results from intervention scale up- not allowing access interventions to overly impact mCPR growth in places where the potential use gap is small and increasing demand when SBC and other demand side interventions are scaled up. This concept can also be useful to help donors, national governments, and implementers more strategically prioritize investments.

## Data availability

The Demand Curve was developed using publicly available data from Demographic and Health Surveys, which is available from: www.statcompiler.com. The following indicators were extracted for all countries with available data:

- Current use of any modern method of contraception by all women
- Current use of any modern method of contraception by currently married women
- Mean ideal number of children for all women
- Unmet need for family planning, total, for currently married women
- Future use of contraception: Intends to use in the future
- Future use of contraception: in next 12 months
- Future use of contraception: Use later
- Future contraceptive use: Unsure about timing

## Supplementary material

**Supplementary File 1: DHS Data Used for Analysis.**
