# Supplementary material for: The maximum contraceptive prevalence ‘demand curve’: guiding discussions on programmatic investments
Source: Gates Open Res. 2017 Dec 22;1:15. [Version 1] doi: 10.12688/gatesopenres.12780.1 (PMC5771155; doi:10.12688/gatesopenres.12780.1)
Supplement: Peer review history

# Peer review: Referee response for version 1

**DOI:** 10.21956/gatesopenres.13840.r26167

**Published:** 2018-01-15

**Authors:** Ann Biddlecom

**Affiliations:** 1 Guttmacher Institute, New York City, NY, USA

The paper presents a helpful heuristic based on family size desires to guide assessments of the balance of investments for demand generation for contraceptive use and supply-side interventions.

 1. Additional detail on the method is needed to clarify the criteria to set the exponential curve that “best touched” the maximum mCPR values at each level of ideal number of children. The heuristic and interpretation are very different if an exponential curve is fit to the 51 maximum mCPR values for ideal family sizes 3 or greater (e.g., for married and cohabiting women, the formula is

 y = 343.72e-0.592x).

 2. The conclusions about the method and its performance should be qualified by the fact that this heuristic focuses on demand for modern methods of contraception as it relates to ideal family size. The approach does not address two other important components of contraceptive demand that are especially relevant in high-fertility contexts: i) birth spacing (delaying the start of childbearing is less of an issue) and ii) the transition from using traditional methods of pregnancy prevention to modern methods (with evidence of under-reporting of traditional method use in surveys – see Rossier and Corker. 2017. Population and Development Review 43 (S1):192-215).

 For example, birth spacing was a major part of contraceptive demand in the Navrongo community health and family planning experiment, and the form of “supply-side” activities helped address some of the demand-side barriers to modern method use. The 1995 reference included in the paper could be updated with the long-term assessment of the experiment in that “initial effects met the need for child spacing” and that a longer-term fertility impact was not observed, in part because social mobilization efforts were neglected after scale-up (see Phillips, et al. 2012. Studies in Family Planning 43(3):175-90).

 3. The time trends for illustrative countries in Figure 3 were helpful. 

 Minor points:

 4. In the analytical data set, there are 241 observations (DHS) and one RHS observation.

 5. (page 2, para 2) Spell out SBC.

 6. (page 2, para 5) “…or a women…”

 7. (page 5, last para) There is an end note #6 that does not link to anything.

I have read this submission. I believe that I have an appropriate level of expertise to confirm that it is of an acceptable scientific standard, however I have significant reservations, as outlined above.

# Peer review: Referee response for version 1

**DOI:** 10.21956/gatesopenres.13840.r26165

**Published:** 2018-01-09

**Authors:** Philip Anglewicz

**Affiliations:** 1 Department of Global Community Health and Behavioral Sciences, Tulane University School of Public Health and Tropical Medicine (TUSPHTM) , New Orleans, LA, USA

This research examines the relationship between access and demand for family planning, using data from Demographic and Health Surveys for a number of countries. The authors focus on the "demand curve," or the plot of modern contraceptive use and the mean ideal number of children, which they suggest represents the maximum mCPR; and the gap between this curve and actual mCPR, which they call the "potential use gap."

 This research is interesting and carefully-conducted. DHS data are well-suited for this analysis, and ensure that the analysis can easily be reproduced- or expanded upon. Regarding the latter, it would be very interesting to see more about the measurement of this "potential use gap", and potentially other factors that are associated with larger or smaller gaps across countries and over time- which again could be done using DHS data. 

 Overall, in my opinion, this short paper makes a worthwhile contribution to the literature on family planning programs.

I have read this submission. I believe that I have an appropriate level of expertise to confirm that it is of an acceptable scientific standard.

# Peer review: Referee response for version 1

**DOI:** 10.21956/gatesopenres.13840.r26168

**Published:** 2018-01-02

**Authors:** John Cleland

**Affiliations:** 1 Faculty of Epidemiology and Population Health, London School of Hygiene & Tropical Medicine, London, UK

The likelihood that high childbearing desires in sub-Saharan Africa constrain increases in contraceptive use and fertility decline is a familiar theme. Your analysis is an ingenious and innovative attempt to quantify the constraint and classify countries in terms of their potential for future gains in mCPR in the presence of unchanging indicators of demand for fertility regulation. You examined three such indicators: unmet need, intention of non-users to use contraception in the future, and total desired family size. To your surprise, and certainly to mine, total desired size provided the clearest relationship to current levels of mCPR and to future increases in mCPR. The surprise stems partly from a broad consensus that absolute numbers of children desired is the least reliable and valid of all indicators of demand for children and by implication of demand for fertility regulation (see eg Casterline and El-Zeini 2007). It suffers from rationalisation of realised fertility: older women of higher parity typically state desired sizes that are two children higher than young women. It would have been theoretically preferable to represent desired size by the reports of younger women, say aged 20-29. Further problems arise from non-numerical responses (Frye and Bachan 2017). Surprise also stems from the results for sub-Saharan Africa, a region where contraceptive use (and unmet need) is largely generated by a birth spacing or postponement motive, which is very loosely connected to any numerical desired family size target. Indeed some analysts have found that fertility transition in Africa, at least in the early stages, is taking a very different form from those in other regions, dominated by widening birth intervals rather than parity-specific limitation (Moultrie *et al.* 2012).

 Surprising, even counterintuitive, results need particularly rigorous interrogation to be convincing and below I make a suggestion.

1. Perform a sub-analysis for the countries of sub-Saharan Africa because it is only here that demand constraints are an important concern. Women in this region has always expressed much higher desired sizes than women in other regions. Even in the 1970s and early 1980s, mean desired sizes in Asia and Latin America were typically around four children whereas in Africa they were typically seven or eight (Lightbourne 1987). I am dubious that it makes sense to develop the demand curve by pooling results from all DHS countries as they are at very different stages of fertility transition. You accept that the demand curve is really only relevant for countries with high fertility desires and I note that most of the non-African FP2020 priority countries have such low mean desired fertility that demand is not a constraint.
2. Demonstrate at least in an annexe for countries in sub-Saharan Africa that total desired size is more powerfully predictive of future increases in mCPR than unmet need or intention to use. My hunch is that unmet need will perform as well as, if not better than, total desired size. I did a quick check on the magnitude of unmet need for the seven African countries where, based on the demand curve, you deduce that little or no potential for gains in mCPR exist and for the ten countries where you deduce that large gains exist. In the former group unmet need ranges from 10% to 23% with a mean of 19%. In the latter group, it ranges from 19% to 38% with a mean of 29%. The unmet need indicator gives the same signal as the demand curve: the potential for quick gains in mCPR appears to be greater in the group of ten than the group of seven. But the unmet need lens, unlike the demand curve verdict, suggests a considerable potential for increase in mCPR even in the seven countries.

I disagree with the way that you conceptualise demand and supply as independent entities. You write that “changing levels of demand requires intensive interventions aimed at addressing a wide range (of) societal norms, and not just existence of barriers to contraceptive use”. Elsewhere you say that “social norms are often slow to change”. In my view it is more valid to accept that demand and supply interact. Means can influence motives eg the desire for overseas vacations arose from the advent of cheap air travel. In the case of contraception, it seems likely that legitimation of, access to, and uptake of contraceptives will destabilise childbearing aspirations by allowing new possibilities and extending human agency. Radical changes in fertility attitudes usually accompany increases in contraceptive use rather than precede them. The policy lesson that I draw for eg Niger and Chad is similar to yours, namely to develop a much stronger communication component of their family planning programs, though I would continue to give priority to reducing barriers to use. The short term aim in countries with very low mCPR should be to raise the prevalence of satisfied users of modern contraception towards 20% and thus harness the power of social diffusion.

 As for the slow pace of change in social norms, you may be correct in general, but there are instances when childbearing attitudes have changed suddenly. In Kenya the World Fertility Survey of 1979-80 depicted a total desired size of 7.2 children with only 16% of women wanting no more. A vigorous family planning was launched shortly thereafter and by 1989 desired size had fallen to 4.8 and the proportion wanting no more had swollen to 49%. In Rwanda, desired size changed only modestly but, between 2000 and 2007-8, the percent of wanting no more children rose from 34% to 49%, with a similar shift in Malawi between 1992 and 2000 from 25% to 42%. At least in East Africa, abrupt changes in fertility norms are possible. Some consideration of these and related issues in the Discussion would make your paper less mechanical and abstract. You might also note that your emphasis on raising demand runs counter to the current dominant rights-based international rationale for family planning investment which is to satisfy existing demand. It would be wise to anticipate objections from some quarters.

 At the top of the right-hand column of page 5 you note that mCPR may be lower than its potential because couples are finding other ways to regulate childbearing and come close to achieving desires. This is a good point and could be elaborated for West and Central Africa. In some countries of these sub-regions TFRs have fallen substantially with little recorded increase in modern CPR (eg Benin). Ghana and Kenya have similar TFRs but mCPR is twice as high in Kenya (Askew et al. 2017). Moreover in Ghana, elite metropolitan women achieve very low fertility with a lower mCPR than other population segments (Machiyama and Cleland 2015). It appears that women are spurning mainstream modern methods in favour of a combination of periodic abstinence, withdrawal, condoms and emergency contraception, probably with medical abortion as backup (Marston et al. 2017; see also Rossier and Corker 2017). An exclusive focus on mCPR is a limitation.

I have read this submission. I believe that I have an appropriate level of expertise to confirm that it is of an acceptable scientific standard, however I have significant reservations, as outlined above.

## References

[1] AskewIMaggwaNObareF: Fertility Transitions in Ghana and Kenya: Trends, Determinants, and Implications for Policy and Programs. *Population and Development Review*.2017;43(S1) : 10.1111/padr.12010 289-307 10.1111/padr.12010

[2] CasterlineJBEl-ZeiniLO: The estimation of unwanted fertility. *Demography*.2007;44(4) :729-45 1823220810.1353/dem.2007.0043

[3] FryeMBachanL: The demography of words: The global decline in non-numeric fertility preferences, 1993-2011. *Popul Stud (Camb)*.2017;71(2) : 10.1080/00324728.2017.1304565 187-209 10.1080/00324728.2017.1304565 28440109PMC5525551

[4] LightbourneR.E: Reproductive preferences and behaviour. *In The World Fertility Survey: An Assessment. Oxford*.1987;838-861 Reference source

[5] MachiyamaKClelandJ: Unmet need for family planning in Ghana: the shifting contributions of lack of access and attitudinal resistance. *Stud Fam Plann*.2014;45(2) : 10.1111/j.1728-4465.2014.00385.x 203-26 10.1111/j.1728-4465.2014.00385.x 24931076

[6] MarstonCRenedoANyaabaGN: Fertility regulation as identity maintenance: understanding thesocial aspects of birth control. *Journal of Health Psychology (online first)*. 10.1177/1359105317726367 10.1177/1359105317726367 PMC588178928925281

[7] MoultrieTASayiTSTimæusIM: Birth intervals, postponement, and fertility decline in Africa: a new type of transition?. *Popul Stud (Camb)*.2012;66(3) : 10.1080/00324728.2012.701660 241-58 10.1080/00324728.2012.701660 22891624

[8] RossierCCorkerJ: Contemporary Use of Traditional Contraception in sub‐Saharan Africa. *Population and Development Review*.2017;43(S1) : 10.1111/padr.12008 192-215 10.1111/padr.12008 PMC574941429307923
